# Regional-scale management maps for forested areas of the Southeastern United States and the US Pacific Northwest

**DOI:** 10.1038/sdata.2018.165

**Published:** 2018-08-28

**Authors:** Matthew Marsik, Caroline G. Staub, William J. Kleindl, Jaclyn M. Hall, Chiung-Shiuan Fu, Di Yang, Forrest R. Stevens, Michael W. Binford

**Affiliations:** 1 Integrated Data Repository, Clinical and Translational Science Institute and UF Health, University of Florida, Gainesville, FL 32610, USA; 2 Decision Suppor Services, University of Florida Health, Gainesville, FL 32610, USA; 3 International Programs, Institute of Food and Agricultural Sciences, University of Florida, Gainesville, FL 32610, USA; 4 Department of Land Resources and Environmental Sciences, Montana State University, Bozeman, MT 59717, USA; 5 Department of Geography, University of Florida, Gainesville, FL 32611, USA; 6 Land Use and Environmental Change Institute, University of Florida, Gainesville, FL 32611, USA; 7 Department of Geography and Geosciences, University of Louisville, Louisville, KY 40292, USA; 8 U.S. National Science Foundation, Alexandria, VA 22314, USA

**Keywords:** Environmental chemistry, Forest ecology

## Abstract

Forests in the United States are managed by multiple public and private entities making harmonization of available data and subsequent mapping of management challenging. We mapped four important types of forest management, production, ecological, passive, and preservation, at 250-meter spatial resolution in the Southeastern (SEUS) and Pacific Northwest (PNW) USA. Both ecologically and socio-economically dynamic regions, the SEUS and PNW forests represent, respectively, 22.0% and 10.4% of forests in the coterminous US. We built a random forest classifier using seasonal time-series analysis of 16 years of MODIS 16-day composite Enhanced Vegetation Index, and ancillary data containing forest ownership, roads, US Forest Service wilderness and forestry areas, proportion conifer and proportion riparian. The map accuracies for SEUS are 89% (10-fold cross-validation) and 67% (external validation) and PNW are 91% and 70% respectively with the same validation. The now publicly available forest management maps, probability surfaces for each management class and uncertainty layer for each region can be viewed and analysed in commercial and open-source GIS and remote sensing software.

## Background & Summary

Forests cover about a third of land area in the coterminous United States (US), with the largest tracts of forest land managed by the federal government for preservation or multiple uses^1^. The top ten largest private landowners in the United States own and manage forestlands primarily for forest products and, increasingly, for real estate development and investments^2^. Forest management practices (e.g. harvesting methods, planting decisions, prescribed fire and fire suppression, and road building) are continually changing in response to policy, socio-economic conditions, climate, and scientific knowledge^3^.

While the effects of management on forests are well-studied at the stand to larger management units such as a national forest scale^4^, little is known about how socioeconomics, land use, and management decisions influence forest ecology across landscapes larger than national forest boundaries such as regions and continents^4^. Without regional scale knowledge, predictions of changes from climate, land use, or policy on forest structure and function remain uncertain, thereby limiting the evaluation of management scenarios to improve forest resilience and sustainability.

Nearly 41% of US private forests have management plans^5,6^ but assimilating these plans to map management activities is difficult and error-prone due to non-systematic information about mixed management practices. Since many management plans are not spatially referenced they are not conducive for spatial analysis. Mapping the ecological effects of forest management across broad geographic extents remains a challenge^7^, therefore gaps exist in the characterization of spatial patterns in forest extent and types of forest management strategies. Climate, ecological disturbance, and forest management interact to influence ecosystem processes of forests across extensive spatial and temporal scales. However, at present, Earth systems models that examine the effects of environmental change do not sufficiently incorporate ecosystem management^8^ and a consistent and systematic approach to mapping forest management at regional and continental scales is needed^9^.

**Figure 1:**
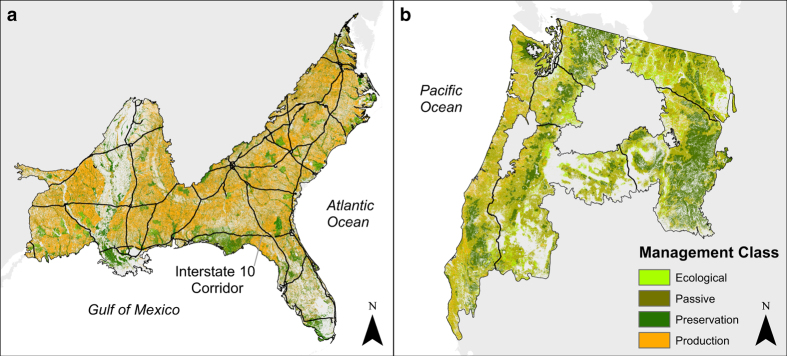
Forest management maps. The SEUS (**a**) and PNW (**b**) forests represent, respectively, 22.0 and 10.4% of forests in the coterminous US. The management maps were created with a random forest classifier using seasonal time-series analysis of 16 years of MODIS 16-day composite Enhanced Vegetation Index, and ancillary data. The SEUS map has an overall accuracy of 89% (10-fold cross-validation) and 67% (external validation) and the PNW map has overall accuracies of 91 and 70%. Raster resolution is 250 meters, and number of forested pixels are n = 9,810,118 for the SEUS and n = 4,638,101 for the PNW. Interstate 10 (I-10) is the southernmost interstate in the SEUS (in black).

We produced forest management maps for two forested regions of the continental United States, the Southeastern Coastal Plain and Piedmont (SEUS) and the Pacific Northwest (PNW), where production is the main management strategy and production cycles are a large cause of land cover change and terrestrial carbon cycles, but different types of land ownership and forest management practices are at play (Fig. 1). The SEUS forest ecosystem is a fire-dominated system with most native trees adapted to short-period events (e.g. 3–5 years^10^). About 85% of SEUS forested land is privately owned, with more than half (54%) owned by corporations^11^. These privately-owned corporate lands are primarily managed for silvicultural production and have an average harvest rotation of 18–20 years. PNW forests ecosystem have adapted to a disturbance regime consisting of wind, fire and beetle disturbance vectors^12^. Two-thirds of forested land is publicly owned and 44% of private forested land is owned by corporations^11^. Areas managed for silvicultural production has an average harvest rotation of about 70 years.

Historically, the interaction of forest policy with land-use and economic priorities has created a mosaic of forest management types in both the SEUS and the PNW. These can be simplified into four management types^9^: 1) production forestry, 2) ecological forestry, 3) passive management, and 4) preservation management. The primary goal of *production forestry* is extraction of wood products for economic gain. Common production forestry silviculture practices include clear-cut harvesting and site preparation with fertilizer and pesticides. *Ecological forestry* aims to balance wood products extraction with maintenance of other forest ecosystem services, such as habitat provision, water resources and carbon storage^12^. Harvests occur periodically with methods like variable retention harvesting performed across decades to maintain an uneven aged forest, a practice which mimics relatively fine scale local disturbances to recreate a shifting mosaic of stand age to maintain structural complexity^12^. *Passively managed forests* are those that are largely left alone apart from occasional harvest driven by economic need or opportunity by the landowner. These include naturally regenerating forests without specific apparent management plans, which often have mixed uses, e.g. hunting or recreation areas. *Preservation management* maintains ecosystems based on historical or natural range of variation for conservation, cultural reasons, recreation and wildlife management. Management practices exclude harvesting but often involve prescribed fire, invasive species removal, and the planting of species to manage ecosystem composition.

## Methods

We built a random forest (RF) classifier^13^ to classify management types using a combination of trends, seasonality, and phenological pattern derived from the Breaks For Additive Seasonal and Trend (BFAST) algorithm analysis^14^ of the MODIS EVI. Ancillary covariates such as road density and forest ownership type coupled with expertly classified training samples.

### MODIS Data

We used a 16-year time series (February 2000 to December 2015) of EVI collected by the MODIS Terra satellite platform. The MOD13Q1 data product is a 16-day composite (23 images per year) imaged at 250-meter spatial resolution^15^, resulting in 360 individual EVI images stacked to create a data cube (i.e. EVI time series).

Poor quality pixels, affected by cloud and processing errors, were identified in the MODIS Quality Assurance (QA) VI Usefulness layer. These data were used with a threshold of 0000-0100 (MOD13Q1 vegetation index quality bits 2-5) in the upper half of the quality range^15^. If, within the 360-band EVI data cube, we had more than 75% good data, we set the value of poor quality pixels to ‘missing,’ and linearly interpolated between the two nearest good pixels. Otherwise the pixel was left as no data. We spatially subset the EVI and QA data-cubes to the SEUS and PNW level-3 ecoregions^16^ (Fig. 1).

### Break Detection, Spectral Entropy and Time Series Decomposition

We used the BFAST algorithm^14^ to decompose the EVI data cube into summary data describing trends, seasonality, and breaks in phenological pattern. The BFAST algorithm as implemented in the R system for statistical computing^17^ (R) decomposes a time series into trend, seasonal, and noise components and detects abrupt change, or ‘breaks’ in the seasonal and trend components, which correspond to disturbances, anthropogenic and natural^14^.

Due to the large size of the EVI data cube and large number of forested pixels (9,810,118 for the SEUS and 4,638,101 for the PNW) we ran the BFAST analysis on the HiPerGator High Performance Computing research cluster at Research Computing, University of Florida totaling six weeks of wall time and approximately 54,000 processing hours.

The summary statistics and break locations extracted from the BFAST summary data provided information on the frequency, timing, magnitude and direction of change occurring within the trend and seasonality components of the EVI time series (Table 1). These statistics describe the input EVI signal, the BFAST-derived seasonal, trend, and noise components to define a set of variables used in subsequent analyses. We also calculated spectral entropy^18^, which is a measure of time series complexity related to the number of unique sine/cosine wave series derived from a Fourier decomposition.

### Additional Covariates

In addition to the BFAST summarized covariates and spectral entropy we used five other covariates for the random forest classifier to further describe the forested landscape. We created a 250-meter road density raster using OpenStreetMap data^19^ with the ArcGIS Linear Density tool specifying a 1-km search radius.

Forest ownership data sources from federal and nongovernment agencies were integrated for landowner type (Table 2). Six types of public ownership were identified: federal protected, federal, state protected, state, military, and local; and four types of private ownership: nongovernment organization, private, family, and corporate. The U.S. Protected Areas Database (PADUS) was the primary source for public ownership and U.S. Department of Agriculture (USDA) Forest Service for private ownership (Table 3). The USDA Forest Service defines private ownership across the coterminous United States as family, including individuals; corporate; and other private (includes conservation and natural resource organizations, unincorporated partnerships and associations, and Native American tribal lands). The spatial distribution of private ownership was modelled using Forest Inventory and Analysis (FIA) data^20^. Additional ownership data were cross-walked to the PADUS and USDA ownership based on management goals, skills, budgets, and interests of landowners (Table 3). Overlay analyses and manual editing rectified polygon topology problems (e.g. intersection, separation, and interlacing) to maintain spatial consistency. Public and private ownership were combined through raster processing operations to produce a 250-meter spatial resolution raster data depicting forest ownership.

For the PNW only, we created a thematic raster covariate to represent National Forest Lands with:

- Nationally Designated Management and Use Limitations (https://data.fs.usda.gov/geodata/edw/edw_resources/shp/S_USA.OtherNationalDesignatedArea.zip),
- National Forest System Roads (https://data.fs.usda.gov/geodata/edw/edw_resources/shp/S_USA.RoadCore_FS.zip),
- Roadless Areas (https://data.fs.usda.gov/geodata/edw/edw_resources/shp/S_USA.RoadlessArea_2001.zip,
- National Wild and Scenic Rivers (https://data.fs.usda.gov/geodata/edw/edw_resources/shp/S_USA.WildScenicRiver.zip) and,
- Wilderness boundaries (http://www.wilderness.net/GIS/Wilderness_Areas.zip) for U.S. National Forest Service, U.S. Fish and Wildlife Service, U.S. Bureau of Land Management, and U.S. National Park Service.

We created proportion conifer and proportion riparian spatial data from the Landfire Existing Vegetation Type (EVT) data^21^ by querying “Conifer” and “Riparian” vegetation types to upscale the proportion of the 30-meter spatial resolution of Landfire data to that of the 250-meter resolution of the EVI data cube. This conceptually simple spatial cross-tabulation analysis proved computationally difficult to implement at regional extents due to the large raster sizes of 59,384,315 (9,815,810) pixels for the SEUS and 19,419,379 (4,643,335) pixels for PNW, at 30- (250-) meter spatial resolution. We overcame the memory limitations of running a spatial cross tabulation analysis in GIS software using the ArcGIS *arcpy* Python library to convert the raster data to tables to import to in PostgreSQL open-source Object-Relational database management system and perform the cross tabulation analysis using a sequence of SQL queries.

### Training Sample Development

We created our training dataset using expert opinion and a modified Delphi method^22^. The USFS Forest Inventory Analysis (FIA) dataset would have been an excellent alternative if it were not already an input to the ED2 ecosystem model. Using them to validate the forest management maps would have introduced collinearity among input variables and biased subsequent ED2 model estimates. We also chose against using the FIA data because this would have required a classification of the dataset to fit our four-category categorization of forest management, a process that was beyond the scope of this study. To develop our training dataset, we placed 1000 spatially random points in forested areas in both the SEUS and PNW. Five experts (i.e. remote sensing specialists, forest ecologists, and ecosystem modellers) for the SEUS and two people for the PNW, examined each point with Google Earth, using historical imagery back-catalogue when needed, and designated one of four forest management types using expert knowledge and a rubric for each region. Landfire EVT (Physiognomy and Group Name), ownership (from PADUS and USDA ownership), Landfire Disturbance Type, and Monitoring Trends in Burn Severity were added to each test point to aid the classification of each management type. Regardless of region, points with complete or majority consensus (80%) were assigned the corresponding management type. Sites without majority agreement were discussed collaboratively, whereby the opinion of an expert could be changed by logical arguments from other participants. New consensus points were then assigned a management type. The management types of unresolved points were designated by regional forest experts. Points for which consensus was not reached were dropped from the training set (i.e. n = 22 for the SEUS, and n = 5 for the PNW).

### Management Type Classification

The RF algorithm grows many classification “trees,” which are decision trees based on thresholds (explained below in fifth paragraph of this section) in the covariate values, each of which produces a classification, as well as “votes” for that class^13^. The algorithm then chooses the classification having the most votes compared to all “trees” in the forest. To classify a new object (a pixel) from an input set of covariates and training data, the input data are passed down each tree in the forest.

A bootstrap sampling is performed on a training set chosen *n* times with replacement from all available training data, *N*. Given the full set of input covariates, a much smaller subset of covariates is randomly selected, at each node in the tree and the best split based on the subset is used as the resulting node split to retain the most information content from the full set of covariates. The RF parameter *mtry*, is the size of the subset and is held constant during the forest growing whereby each tree in the forest is grown to the largest extent possible without pruning. The forest error rate depends on the correlation between any two trees in the forest, increased correlation increases overall error, and the strength of each individual tree in the forest. A low forest error rate depends on the low correlation among trees, and the increased strength of individual trees, denoting a tree is a strong classifier.

The Mean Decrease in Accuracy is estimated during the out-of-bag (OOB) error calculation phase of the RF algorithm. For each tree in the RF the held-out sample observations (those that are OOB) have predictions compared with and without randomly permuting the values for each covariate. The number of votes for the correct class using permuted data is subtracted from the number of correct votes from unpermutated data.

The spatial covariates were sampled and used to classify management into four forestry management classes. Despite a low number of training points for ecological management in the SEUS and ecological and production management in the PNW, we are confident in the performance of the RF algorithm, which is robust against unbalanced sets of training data^23^ and we weighted these underrepresented classes in the initial tuning of the RF model. We removed non-forest pixels, identified from a composite of years 2001-2006-2011 from National Land Cover Dataset (NLCD)^24,25^, from the estimation process and only those MODIS pixels that were 50% or more forest and 50% or more of one of the disturbance/intensity classes were used as training data.

We fit a full random forest model with 500 individual classification trees and seven (7) covariates tried at each split as determined to be optimal by the tuneRF() algorithm in the *randomForest* R package^26^ and post-fitting diagnostics guided the creation of a fitted random forest model used for predictions. We ran an iterative classification to remove covariates that contributed a negative increase in node purity to remove computation burden during the prediction stage. We then selected the 15 most important covariates based on decreased mean accuracy to create spatial predictions using the fitted random forest model.

### Code Availability

The BFAST and RF codes, used to produce the forest management datasets, are publicly available through the figshare repository (Data Citation 1). The code consists of sets of Python (version 2.7) and sets of R (version 3.1 and higher) programming language scripts that must be run sequentially in the following order: 1) 01 MODIS Data Download Preparation.zip (R); 2) 02 Calculate proportion riparian and conifer from Landfire.zip (python); 3) 03 Running BFAST on High Performance Cluster using Moab.zip (R); 4) 04 Random forest classification.zip (python and R). Each script is also internally documented in order to both explaining its purpose (including a detailed description of the GIS-specific spatial operations that it performs) and, when required, guiding the user through its customization.

## Data Records

The forest management rasters and management class probability rasters are available for each region as georeferenced GeoTIFF rasters with 250-meter resolution from PANGAEA (Data Citation 2). They can be download as 7-Zip archives (7-Zip.org). Table 4 details the specifics of each available dataset. The uncertainty layers have a horizontal resolution of 10 kilometres to match the use of the management data sets with a convenient grid cell size of the ED2 ecosystem model^27^. All data are viewable and analysable in commercial and open-source GIS and remote sensing software (e.g. ArcGIS 10× , ENVI 5× , ERDAS Imagine, QGIS 2× , GRASS GIS 7× ) and the raster package in R. All data are in the Albers Conic Equal Area projection (EPSG 5070), NAD 1983 datum and horizontal units in meters.

The forest management rasters (Fig. 1) contain the four categories of ecological, passive, preservation and production management types represented as numerical integers (Value field) and lexical description (Management field). The values in the probability rasters for each management class range from 0 (least likely to be the respective management class) to 1 (most likely). The uncertainty rasters depict the Bayesian simulated proportion^28^ of forest management type at 250 meter resolution within a 10 kilometre cell.

## Technical Validation

### Validation

We specified a ten-fold cross validation internal to the random forest classifier and assessed the individual contribution from each covariate to the overall accuracy of the management maps. The ten-fold cross validation randomly splits data into ten partitions, with model fitting using nine partitions, and model testing using one partition. The procedure is repeated ten times to generate the sample error as an average of the ten validation runs. This bootstrap method provides unbiased estimation of classification errors. We specified an external validation withholding approximately 20% of data from the training set described above. For each region 178 (SEUS) and 194 (PNW) of the 1000 points were omitted from the training set and used for external error analysis. We constructed an error matrix from the OOB data and external validation data, and calculated commission, omission, and overall errors.

The SEUS map (Fig. 1a) has an overall accuracy of 89% for the 10-fold cross-validation or 67% for the external validation in Table 5 and the PNW map (Fig. 1b) has overall accuracies of 91% and 70%, respectively (Table 6).

### Uncertainty Analysis

To assess uncertainty when upscaling the forest management maps from 250-m spatial resolution to the 10-km grain of ED2 (ref. 27) we performed a Bayesian analysis proposed by Quaife et al.^28^ to model uncertainty in categorical maps aggregated to coarser spatial resolutions. The analysis calculates the posterior distribution of true management classes coupling the observed proportion of management classes in each 10-km site with a confusion matrix produced from the validation methods above, using Monte Carlo simulation to sample the posterior distribution. We considered only forested pixels within each 10-km site and used the standard deviation from the Bayesian analysis to represent uncertainty.

**Figure 2:**
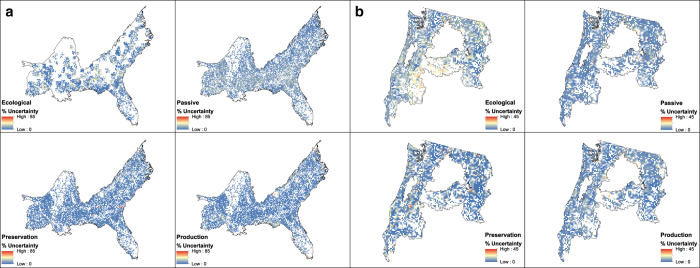
Uncertainty maps of each forest management class. Uncertainty maps, expressed as a percentage, for the SEUS (**a**) and the PNW (**b**) were calculated using the mean and standard deviation posterior distributions of modeled proportions from Bayesian analysis of 250 m MODIS cells within each 10 km ED2 cell.

**Figure 3:**
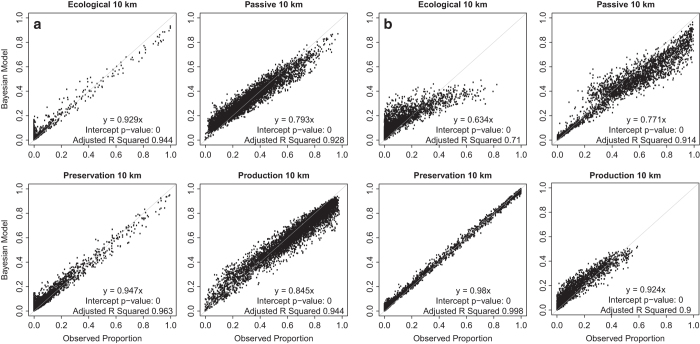
Scatter plots of land cover proportion. Forest management maps for the SEUS (**a**) and the PNW (**b**) indicate fit between the Bayesian modeled and observed proportions of the 250 m forest management cells within each 10 km ED2 cells.

The uncertainty analysis quantified the amount of error when scaling the management map from 250-meter resolution to the 10-km aggregate resolution for use by ED2 (ref. 27) by modelling the proportion of each management type as they fit inside an ED2 10-km site. The maps (Fig. 2) give us the spatial distribution of uncertainty. Scatterplots of the observed proportions and the mean of the samples from posterior distribution (Fig. 3), while ignoring the spatial component, indicate fit between the Bayesian modelled and observed proportions. Recall the 978 training points of the SEUS were sub-divided into 800 for training (used for 10-fold cross-validation in the random forest model) and 178 for external validation, hence the two confusion matrices.

In general, there is low uncertainty in the classification of forest management. (Fig. 2), however, the results from the Bayesian analysis of proportions are slightly biased at lower and higher values of the proportion of a forest management type within 10-km cells (Fig. 3). The plots from the random forest model show tighter fit to the 1:1 line (i.e. where the RF algorithm closely models the training data), while those from the external validation show greater spread and under-prediction bias at larger values of observed proportions. The under-prediction is noticeable also in the plots from the RF model (Fig. 3). We see a higher amount of uncertainty associated with peripheral pixels for each forest management type. That is, we are more certain about the forest type of the core area of a forest patch compared to the edge of the forest patch regardless of forest management type. Note the relative ‘high’ uncertainty for passive and production classes north of the highway I-10 corridor and Gulf of Mexico coast is possibly due to edge effects and/or lower total pixel counts.

In the PNW, we are most certain about our ability to classify preservation and production management. We have low confidence in the classification of ecological management, which could be because of small sample size despite the weighting during the tuning of the initial RF model. The evidence of low adjusted r-squared (0.63) from comparing the Bayesian simulation results with the observed proportion (Fig. 3) indicates that our predictions of the higher proportions of ecological forestry are not as certain as other management types. In the SEUS preservation and ecological forestry show lowest uncertainty based on adjusted r-squared of the fit between the observed proportions and those from the Bayesian analysis. Classification of passive management is the most problematic at high observed proportions, possibly because passively managed patches are probably smaller.

## Usage Notes

We successfully mapped forest management in both the Southeastern U.S. coastal plain and Piedmont (SEUS) and in the U.S. Pacific Northwest forest area (PNW) using satellite derived data and expert classified training samples. Once we determined the suite of covariates and algorithm parameters from our classification of a pilot single Landsat footprint, we created forest management maps of the entire Southeastern U.S. coastal plain and Piedmont (SEUS) and in the U.S. Pacific Northwest forest area (PNW) (Fig. 1) for the composite time period 2000-2015. We mapped four management classes of ecological, passive, preservation and production management^9^ that collectively represent 59.29% of the SEUS of the total land cover (1,034,633. km^2^) and 62.80% of the PNW land cover (461,593.8 km^2^), and representing, respectively, 22.0% and 10.4% of the coterminous U.S. forests.

### General Usage

These maps are a first attempt, as far as we know, to map forest management at regional extents in the United States. Land cover is a fundamental variable that impacts and links many parts of link between the human and physical environments. Land use is the socio-economic intent or purpose behind the management of the land surface (e.g. residential, commercial, parks and green spaces). Land cover is the biophysical covering of the land surface (e.g. forest, grassland). Changes in observed land cover patterns are the net result of individual, communal or societal decision-making processes regarding the relative returns to land use^29^ set within a local, regional or national context. Hence, land cover, along with pattern analysis and social science measures, can be used to infer the changing patterns in land use (i.e., to link land cover to land use).

The forest management maps have a spatial resolution of 250 m and results from an analysis of a composite of phenological patterns and changes in the patterns from February 2000 through December 2015. The maps represent a temporal composite of the 16 year time period to be used as dominant forest management conditions during this time range. These maps were created originally to represent the proportion of management classes and to parameterize forest functional types for the ecosystem dynamics simulation model ED2 (ref. 27) to simulate carbon cycling at a 10-km spatial resolution. The development of a forest management functional type framework allowed the addition of forest management practices to Earth systems models. The many varied management practices can be grouped into regionally specific sets of practices. These management functional types include a variety of approaches including the short rotation times, clearcuts, and even-age stands used by production forestry systems and the uneven aged stands with selective harvesting used by ecological forestry systems. In practice, the forest management data can be used in any ecosystem simulation model that requires explicit spatial representation of forest management classes and forest management functional types.

Following Becknell et al.^9^, we established four simplified categories of forest management. However, because production forestry and ecological forestry possibly represent endpoints along a gradient of production-based silviculture practices, they were difficult to clearly distinguish thematically in all cases. This gradient includes even-age stand management; clearcutting, coppicing (i.e. overstory removal), seed-tree, shelterwood and, uneven-age stand management; patch or group selection, thinning, and single tree selection^30^. Many of these silvicultural approaches can be readily observed through remote imagery. For instance, our classification of production forestry is based on extraction of wood products for economic gain and that can be easily applied to clearcut sites as well as some other even-age management applications. However, our classification of ecological forestry as one that mimics relatively fine scale disturbances to recreate a shifting mosaic of stand age may be harder to distinguish via remote sensing from shelterwood or patch selection timber harvest approaches. Most likely the gradient between production and ecological forestry practices would require a fuzzy logic rule set to reduce classification error^31^. Additionally, ecological forestry occur on lands that require follow-up management post-harvest. Franklin et al.^12^ defines ecological forestry as a “is a three-legged stool.” Where the legs, or principles for management, include (1) retention of biological legacies at harvest; (2) intermediate treatments that enhance stand heterogeneity; and (3) allowances for appropriate recovery periods between regeneration harvests.

We acknowledge that mapping the SEUS and PNW regions of the United States may at first appear to limit user applications, however, we posit the maps and underlying regional-scale datasets produced to date represent a salient and overdue contribution to the ecosystem modelling community in the US. The SEUS and the PNW forest regions have the largest total area of forest, compared to the other US forest regions^32^ with the largest area of forested timberland in the SEUS and the highest are of forested reserves in the PNW. This project concerns the major ownership types and management styles that occur in the US, therefore we began this project by looking the regions with large areas of forest and large proportions of active forested timberland, being harvested and replanted, and under various ownership types including private, private corporate, and reserved forest. We appreciate the potential for mapping forested lands over the whole of North America and endeavour to do so in a subsequent phase of this project.

All maps contain errors and in thematic maps the nature and extent of misclassifications are addressed in accuracy assessments through the use of a confusion matrix. The confusion matrices (Tables 5 and 6) were produced at a regional scale, appropriate for these maps. It is becoming more common to construct confusion matrices against higher resolution, manually interpreted satellite data^28^. However, Fang et al.^33^ noted confusion matrices developed at fine scales might have much different error rates than regional matrices. The confusion matrix also has its own suite of inherent uncertainties as the reference data itself can also contain unmeasured sources of error^34^. Additionally, although a confusion matrix is excellent at capturing thematic errors of omission and commission, it cannot capture all the non-thematic error that affects classification^35^. Ultimately, obtaining a reliable confusion matrix and associated indices can be problematic^36^. However, it currently remains the core accuracy assessment tool^34^ and the map user is limited to the data provided unless they conduct their accuracy assessment^37^.

The SEUS silviculture harvest occurs on a 18–25-year rotation and we have satellite imagery that nearly reaches that time frame, hence, the SEUS mapping effort captures most silvicultural activities. The PNW as harvest rotations are much longer (e.g. 40–60 years^38^) leading to forest harvest activities that extend beyond the period of our remote sensing data. Interpreting silvicultural activities that occurred before our period of record offered a challenge to our mapping effort (i.e. we could not discern when a patch of secondary growth forest was harvested initially). For instance, we only considered pixels mapped as forest in the 2011 NLCD in our analysis; however, PNW clearcuts mapped as non-forest in the 2001 and 2006 NLCD were omitted from our analysis resulting in some misclassification. For the PNW we suggest an alternative approach to this problem below.

### Misclassifications: NLCD and PNW Clearcuts

The treatment of clearcut areas in the PNW as non-forest by NLCD is caused misclassification errors. One solution to this problem may be that the NLCD has a 2001–2011 from-to change index, most useful because it can represents succession from clearcut to forest or land use change from forest to clearcut. In other places forest to grass, grass to shrub, or shrub to forest is captured when there was a clearcut. NLCD should be used in conjunction with other vegetation transitions maps to capture the succession pathways of forest cover. For example, the Landfire Vegetation Transition Magnitude data product (https://www.landfire.gov/vtm.php) captures clearcuts and conversions from forest to pasture, agriculture, and urban reasonably well like NLCD. To map omitted clearcuts, we recommend using either the Landfire Vegetation Transition Magnitude and/or the NLCD 2001-2011 from-to product to extend the mapped forest area with the following rule set.

It is a forest if:

- It is forest cover in Landfire 2001, 2006 or 2011,
- It was forest cover in NLCD then transitioned to grass, or
- Transitioned from grass to shrub or shrub to forest.

### Recommendations for improved mapping methods

We recommend better spatial and temporal resolution of ancillary data (e.g. roads, ownerships, USFS management) and incorporation of local scale management plans into creation of management maps. We need better spatial and temporal representation of forest ownership. Private ownership is especially unclear when working across state boundaries as no two states have spatial and attribute consistency between tax parcel datasets. We need temporal snapshots of management maps since we developed only a composite of management aggregated from 15 years of MODIS EVI data. This is challenging as training data are needed for each time period of interest, and we would need to fully or partially automate the training methodology (e.g. image segmentation and recognition, and machine labeling of training data).

The Google Earth time slider was used to estimate the dominant forest management conditions to train the RF classifier. While it provides a retrospective view of historic forest cover conditions and spatial patterns, the time slider has limitations. The intervals of the time slider were not consistent over the two regions and are dependent on the available imagery in the Google archives. For example, imagery exists approximately every year between 2004 and 2017 for a forest tract northeast of Gainesville, Florida, while imagery of forest southeast of Hattiesburg, Mississippi is available every two to four years for the same period. Additionally, the spatial resolution and color depth varies across both regions. It is very difficult to compile a temporally consistent and high spatial resolution set of images to derive the training samples needed for the random forest classifier; hence Google Earth imagery is an attractive alternative.

The maps represent a temporal composite of forest management. To truly capture forest dynamics as they affect the carbon cycle, ideally, we would create an annual time series of management maps. Given the labor-intensive process of manually coding 1000 training samples for each region, while desirable, a time series of annual forest management conditions was prohibitive to create and beyond the scope of this effort. Automated image recognition coupled with machine learning could speed up the development of training samples and allow for repeated characterization of management practices over time.

## Additional information

**How to cite this article**: Marsik, M. *et al*. Regional-scale management maps for forested areas of the Southeastern United States and the US Pacific Northwest. *Sci. Data* 5:180165 doi: 10.1038/sdata.2018.165 (2018).

**Publisher’s note**: Springer Nature remains neutral with regard to jurisdictional claims in published maps and institutional affiliations.

## Supplementary Material



## Figures and Tables

**Table 1:** BFAST summary variables.

**BFAST VARIABLE**	**DESCRIPTION**	**SEUS**		**PNW**	
		**MIN**	**MAX**	**MIN**	**MAX**
bd_b	location of break with the biggest decrease between current and previous trends	46	314	46	314
bd_b_diff	largest decrease in difference between break in trend and previous trend	−6148.36	4446.837	−5245.37	3453.825
bd_b_inqtrng	largest decrease in variability between 25th and 75th quartiles between location of declining break and previous trend	2	132	2	133
bd_b_mean_diff	largest decrease in the difference of mean EVI value between break in trend and previous trend	-4495.99	2837.041	-4311.45	2642.348
bd_sb	location of break with the biggest decrease in the seasonal component	46	314	46	314
bd_sb_entropy_diff	largest decrease of the difference in entropy detected in the break in the seasonal component	−0.521	0.515	−0.549	0.478
bd_sb_inqtrng	largest decrease in variability between 25th and 75th quartiles between location of declining break and previous trend in the seasonal component	2	41	3	41
bd_sb_range_diff	largest decrease in the difference of the EVI value range between declining break and previous trend in the seasonal component	−4630.4	6079.135	−5675.56	4652.122
bi_b	location of the break with the biggest increase between current and previous trends	46	314	46	314
bi_b_diff	largest increase in difference between break in trend and previous trend	−4847	5115.445	−5120.05	4116.391
bi_b_inqtrng	largest increase in variability between 25th and 75th quartiles between location of recovery break and previous declining trend	2	132	2	133
bi_b_mean_diff	largest increase in the difference of mean EVI value between break in trend and previous trend	−4020.48	3896.045	−3879.1	3348.371
bi_sb	location of break with the biggest increase in the seasonal component	46	314	46	314
bi_sb_entropy_diff	largest increase of the difference in entropy detected in the break of the seasonal component	−0.518	0.515	−0.549	0.528
bi_sb_inqtrng	largest increase in variability between 25th and 75th quartiles between location of recovery break and previous declining trend in the seasonal component	2	41	3	41
bi_sb_range_diff	largest increase in the difference of the EVI value range between declining break and previous trend in the seasonal component	−4584.58	5979.124	−5537.2	6075.467
detected_breaks	number of detected breaks in trend component	0	3	0	3
detected_breaks_seasonal	number of detected breaks in seasonal component	0	3	0	3
entropy	entropy in time series	0.246	0.942	0.302	0.96
entropy_seasonal	entropy in seasonal component	0.022	0.664	0.019	0.732
lb_b	location of the longest break	46	314	46	314
lb_b_diff	largest increase in difference between break in trend and previous trend	−5767.18	4446.837	−5120.05	3363.041
lb_b_inqtrng	variability between 25th and 75th quartiles between location of longest break and previous trend	2	132	2	133
lb_b_mean_diff	location of mean difference of longest break from previous trend	−4020.48	3292.601	−3879.1	3348.371
lsb_b	location of the longest break in the seasonal component	46	314	46	314
lsb_break_num	number of the longest break in the seasonal component	1	3	1	3
lsb_sb_entropy_diff	difference in entropy of the longest break in the seasonal component	−0.521	0.515	−0.549	0.528
lsb_sb_inqtrng	variability between 25th and 75th quartiles between location of longest break and previous trend in the seasonal component	2	41	3	41
lsb_sb_range_diff	difference of the value range of the longest break in the seasonal component	−4312.92	5614.436	−5537.2	6075.467
The summary statistics and break locations extracted from the BFAST summary data provide information on the frequency, timing, magnitude and direction of change in the Enhanced Vegetation Index (EVI) occurring within the trend and seasonality components of the EVI time series. These statistics describe the input EVI signal, the BFAST-derived seasonal, trend, and noise components to define a set of variables used in random forest classification.					

**Table 2:** Input data sources for ownership.

**Data source**	**Owner type**
USGS Protected Areas Database of the United States (PADUS)	Federal, State, Local Government, and private http://gapanalysis.usgs.gov/padus/
NCED	Federal, Tribal, State, Regional agency, Local Government, Non-Governmental Organization (NGO), Private http://conservationeasement.us/
Military installations, Ranges, and Training Areas, Acquisition Technology and Logistics	Military
	https://catalog.data.gov/dataset/military-installations-ranges-and-training-areas
US Military Bases, Bureau of Transportation Statistics	Military https://koordinates.com/layer/749-us-military-bases/
Bureau of Land Management - Surface Management Agency	Bureau of Land Management https://catalog.data.gov/dataset/blm-national-surface-management-agency-area-polygons
Federal Lands of the United States, USGS	DOD, FS(national Forest), FWS (national wildlife refuge system), NPS(national park system), Other, TVA (Tennessee Valley Authority)https://nationalmap.gov/small_scale/mld/fedlanp.html
Public and private forest ownership in the conterminous United States: distribution of six ownership types, USDA	Federal, State, Local, Family, Corporate, Other private (This dataset was only used for private area owner type classification) https://www.fs.usda.gov/rds/archive/Product/RDS-2017-0007
Forest ownership data sources from federal and nongovernment agencies were integrated for landowner type. Six types of public ownership were identified: federal protected, federal, state protected, state, military, and local, and four types of private ownership: nongovernment organization, private, family, and corporate.	

**Table 3:** Cross walk of owner types standardized to the PADUS and USDA ownership based on management goals, skills, budgets, and interests of landowners.

**Owner Type**	**NCED**	**Bureau of Land Management**	**Federal Lands of the United States, USGS**	**USDA public and private ownership US**
Federal protected			FWS, NPS	
Federal	Federal, Regional agency	Bureau of Land Management	FS, Other, TVA	
State protected				
State	State, Regional agency			
Military			DOD	
Local	Local Government			
Nongovernment organization	NGO, Tribal, Regional agency			Other private
Family				Family
Corporate				Corporate
Private	Private			
Overlay analyses and manual editing rectified polygon topology problems (e.g. intersection, separation, and interlacing) to maintain spatial consistency. Public and private ownership were combined through raster processing operations to produce a 250-meter spatial resolution raster data depicting forest ownership.				

**Table 4:** Datasets available for download.

**Dataset Name**	**File Size (Mb)**	**Dimensions (columns, rows)**	**Attributes**
PNW Forest Mgmt Map	1.62	4118, 4562	Value, Management
PNW Probability of Ecological	19	4118, 4562	
PNW Probability of Passive	22.1	4118, 4562	
PNW Probability of Preservation	17.8	4118, 4562	
PNW Probability of Production	17.6	4118, 4562	
PNW Uncertainty Ecological	0.046	103, 115	
PNW Uncertainty Passive	0.046	103, 115	
PNW Uncertainty Preservation	0.046	103, 115	
PNW Uncertainty Production	0.046	103, 115	
SEUS Forest Mgmt Map	3.72	7487, 6784	Value, Management
SEUS Probability of Ecological	29.02	7487, 6784	
SEUS Probability of Passive	48.4	7487, 6784	
SEUS Probability of Preservation	33.3	7487, 6784	
SEUS Probability of Production	49	7487, 6784	
SEUS Uncertainty Ecological	0.125	188, 170	
SEUS Uncertainty Passive	0.125	188, 170	
SEUS Uncertainty Preservation	0.125	188, 170	
SEUS Uncertainty Production	0.125	188, 170	
PNW Validation Points	0.06		MGMT_REF, MGMT_PRED
SEUS Validation Points	0.04		MGMT_REF, MGMT_PRED
PNW Training Points			
SEUS Training Points			
pnw_bfast_stack.tif	266	4118, 4562	
seus_bfast_stack.tif	601	7487, 6784	

**Table 5:** Confusion matrices, and producers, users and overall accuracy of the SEUS forest management classification.

	**10-fold cross-validation (n=800)**							**external validation (n=178)**						
			**Ecological**	**Passive**	**Preservation**	**Production**				**Ecological**	**Passive**	**Preservation**	**Production**	
	**Total**	**%**	**26**	**217**	**76**	**481**	**Producers Accuracy**	**Total**	**%**	**2**	**43**	**22**	**111**	**Producers Accuracy**
Ecological	28	3.5	25*	1	1	1	0.89	3	1.69	1*	0	2	0	0.33
Passive	230	28.75	0	188*	4	38	0.82	49	27.53	0	24*	3	22	0.49
Preservation	76	9.5	1	3	66*	6	0.87	20	11.24	0	4	13*	3	0.65
Production	466	58.25	0	25	5	436*	0.94	106	59.55	1	15	4	86*	0.81
	800	Users Accuracy	0.96	0.87	0.87	0.91	0.89	178	Users Accuracy	0.50	0.56	0.59	0.77	0.62
Confusion matrix for the 10-fold internal validation (a) and the external validation (b). Internal validation resulted from the out-of-bag (OOB) error during the training of the random forest classifier. The external validation was conducted with 178 training samples omitted from training of random forest model. The percentage column provides the distribution of validation points among the management classes. The diagonal represents the correctly classified classes with an *. The column with the producers accuracy (i.e. errors of omission) depicts the number of correctly classified management class (on diagonal) divided by the column total. The row with user’s accuracy (i.e. errors of commission) depicts number of correctly classified wound classes (on diagonal) divided by the row total.														

**Table 6:** Confusion matrices, and producers, users and overall accuracy of the PNW forest management classification.

		**10-fold cross-validation (n=800)**						**external validation (n=194)**						
			**Ecological**	**Passive**	**Preservation**	**Production**				**Ecological**	**Passive**	**Preservation**	**Production**	
	**Total**	**%**	**62**	**411**	**201**	**126**	**Producers Accuracy**	**Total**	**%**	**6**	**123**	**47**	**18**	**Producers Accuracy**
Ecological	77	9.63	57*	17	0	3	0.74	18	9.28	1*	15		2	0.06
Passive	389	48.63	4	364*	6	15	0.94	98	50.52	4	83*	3	8	0.85
Preservation	203	25.38	0	8	195*	0	0.96	48	24.74		5	43*		0.90
Production	131	16.38	1	22	0	108*	0.82	30	15.46	1	20	1	8*	0.27
	800	Users Accuracy	0.92	0.89	0.97	0.86	0.91	194	Users Accuracy	0.17	0.67	0.91	0.44	0.70
The 10-fold internal validation (a) and external validation (n = 194) (b) follow the same explanation given in Table 5.														
